# Validation of the Edinburgh postnatal depression scale (EPDS) for screening of major depressive episode among adults from the general population

**DOI:** 10.1186/s12888-014-0284-x

**Published:** 2014-10-08

**Authors:** Alicia Matijasevich, Tiago N Munhoz, Beatriz Franck Tavares, Ana Paula Pereira Neto Barbosa, Diego Mello da Silva, Morgana Sonza Abitante, Tatiane Abreu Dall’Agnol, Iná S Santos

**Affiliations:** Departament of Preventive Medicine, School of Medicine, University of São Paulo, São Paulo, Brazil; Postgraduate Program in Epidemiology, Federal University of Pelotas, Pelotas, Brazil; Department of Mental Health, Faculty of Medicine, Federal University of Pelotas, Pelotas, Brazil; Catholic University of Pelotas, Pelotas, Brazil; Centro de Pesquisas Epidemiológicas - Universidade Federal de Pelotas, Rua Marechal Deodoro, 1160, Pelotas, RS CEP: 96020-220 - Caixa Postal 464 Brasil

**Keywords:** Major depressive episode, Validity of tests, Sensitivity and specificity, Adult, EPDS test, Depression

## Abstract

**Background:**

Standardized questionnaires designed for the identification of depression are useful for monitoring individual as well as population mental health. The Edinburgh Postnatal Depression Scale (EPDS) has originally been developed to assist primary care health professionals to detect postnatal depression, but several authors recommend its use outside of the postpartum period. In Brazil, the use of the EPDS for screening depression outside the postpartum period and among non-selected populations has not been validated. The present study aimed to assess the validity of the EPDS as a screening instrument for major depressive episode (MDE) among adults from the general population.

**Methods:**

This is a validation study that used a population-based sampling technique to select the participants. The study was conducted in the city of Pelotas, Brazil. Households were randomly selected by two stage conglomerates with probability proportional to size. EPDS was administered to 447 adults (≥20 years). Approximately 17 days later, participants were reinterviewed by psychiatrics and psychologists using a structured diagnostic interview (Mini International Neuropsychiatric Interview, MINI). We calculated the sensitivity and specificity of each cutoff point of EPDS, and values were plotted as a receiver operator characteristic curve.

**Results:**

The best cutoff point for screening depression was ≥8, with 80.0% (64.4 - 90.9%) sensitivity and 87.0% (83.3 - 90.1%) specificity. Among women the best cutoff point was ≥8 too with values of sensitivity and specificity of 84.4% (67.2 – 94.7%) and 81.3% (75.5 – 86.1%), respectively. Among men, the best cutoff point was ≥7 (75% sensitivity and 89% specificity).

**Conclusions:**

The EPDS was shown to be suitable for screening MDE among adults in the community.

## Background

Depression is one of the most prevalent mental health disorders all around the world. It interferes with interpersonal relationships and affects the performance of everyday activities [1]. Depression is responsible for a considerable amount of health expenditure, with high economic impact at the level of households, firms and governments [2]. The latest Global Burden of Disease study showed that major depressive disorder was the second leading cause of years lived with disability (YLDs) and a major contributor to the burden of suicide and ischemic heart disease worldwide [3].

A study that included eighteen countries and considered a recall period of one year reported the highest major depressive episode (MDE) prevalence in United States, Ukraine and Brazil (8.3%, 8.4% and 10.4%, respectively) [4]. However, the rates of depression in Brazil are variable, due to the adopted instrument to detect MDE, time frame coverage and different sampling methodology. A recent systematic review and meta-analysis of population-based studies reported a prevalence of depressive symptoms of 14%, 1-year prevalence of major depressive disorder of 8% and a lifetime prevalence of major depressive disorder of 17% among Brazilian adults [5].

Individuals that experience depression are more likely to have new episodes of depression throughout life and comorbidity with other mental health disorders such as anxiety, social phobia and other chronic diseases [6-8], situation that is exacerbated in the absence of adequate treatment. Failure to correctly identify depression can not only negatively impact the health of the individuals, but also could result in an inadequate provision of mental health services in the community [9]. In addition to be affected by prejudice and stigmatization, families bear a significant proportion of the economic and social burden of the disease, due to the lack or inefficiency of available mental health services, a situation most frequent among developing countries [10-12].

Standardized questionnaires designed for the identification of depression are useful for monitoring individual as well as population mental health. Characteristics of the instruments such as the number of questions, easy of understanding, good psychometric properties and free access increase their potential applicability both in research and clinical settings. A great deal of questionnaires are currently available to assess depression, however, many are time consuming, complex and designed to be performed by interviewers highly trained in mental health. The most frequent instruments used for screening depression in Brazil are the Beck Depression Inventory (BDI), the “Geriatric Depression Scale” (GDS) and the Patient Health Questionnaire-9 (PHQ-9). Each instrument has been validated in the Brazilian population and has advantages and limitations that have been reported in previous studies [13-15].

The Edinburgh Postnatal Depression Scale (EPDS), a 10-item scale, developed by Cox et al. was originally devised for the identification of postpartum depression disorders [16]. The use of EPDS is favoured because of the ease and speed of its administration. The clinical and epidemiological value of the scale has been confirmed by several validation studies carried out in different countries mostly among women in the postpartum period, with both sensitivity and specificity in the 70-85% range, depending on the cutoff point [17-25]. Although the EPDS was developed for screening depression in women postnatally, it has been shown to be useful in the assessment of women outside the postnatal period and has been validated among men [21,22]. The fact that the EPDS is already known by health professionals due to its use in the perinatal and postpartum period raises the question whether this instrument could be applied to the adult population outside of the postnatal period. In Brazil, the use of the EPDS for screening depression outside the postpartum period and among non-selected populations has not been validated. Thus, the present study aimed to assess the validity of the EPDS as a screening instrument for MDE among adults from the general population.

## Methods

A population-based cross-sectional study was conducted in the urban area of Pelotas, state of Rio Grande do Sul, southern Brazil, between February and June 2012, to assess the health of adolescents, adults and elderly residents. The city of Pelotas has 328,275 inhabitants according to the 2010 Census and its population is predominantly urban (93.3%). A sampling design of two-stage conglomerates with probability proportional to size was used. According to the 2010 Population Census there were 495 census tracts, the primary sampling units. The secondary sampling units were households. All private households with permanent resident as of December 2011 in the 130 census tracts selected were listed. They were then randomly selected by applying probability proportional to size. All residents of selected households with 20 or more years of age were eligible for the study. Individuals who had cognitive or mental disabilities confirmed by the fieldwork supervisor as well as those institutionalized (hospitals, elderly homes, among others) were excluded. Participants were interviewed at home by trained interviewers through the application of a structured questionnaire. Information was obtained on demographic, environmental and socioeconomic variables and work and health-related behaviours.

### Instrument

The EPDS was originally devised for the identification of postpartum depression disorders for use in clinical and research settings. EPDS is a self-administered, 10-item scale; each item has four possible responses from 0 to 3, with a minimum score of 0 and a maximum of 30. The scale expresses the intensity of depressive symptoms over the preceding seven days. All participants completed the EPDS questionnaire. We used a Brazilian version of the questionnaire. Questions were translated into Portuguese, back-translated again into English and tested in a previous study [24]. In contrast to the original self-administered format, questions were posed to individuals by a trained interviewer, as a single block and in the same order as in the original instrument. The decision to pose the questions verbally was due to the fact that an important proportion of participants had little schooling as well as being unfamiliar with self-administered data collection instruments. The administration of EPDS as an interview is accepted by the instrument's authors [16] and has been previously used [17,24].

### Sample for the validation study

The validation study was conducted only among adults (≥20 years). The sample was selected weekly from interviews conducted in the main study. One in three households included in the main study was randomly selected to be included in the validation study sample. The person responsible for selecting the participants was not aware of EPDS results applied in the main study. More than one adult per household was eligible to be included in the validation study. The present validation study was designed to detect sensitivity and specificity ≥80%, with an error of ten percentage points and a significance level at 95%. The acceptable error was compatible with the logistics of the study and considered satisfactory for a validation study. According to these parameters it would be necessary to include around 200 individuals with MDE and 200 normal.

### Gold standard instrument

The Mini International Neuropsychiatric Interview (MINI), validated in a Brazilian population [26], was chosen as the gold standard instrument. The MINI was designed to be used both in clinical practice and in epidemiological studies and evaluates the presence of mental disorders according to the Diagnostic and Statistical Manual of Mental Disorders – IV revision (DSM-IV) and the International Statistical Classification of Diseases and Related Health Problems – 10^th^ Revision (ICD-10). It was tested against the Structured Clinical Interview for Diagnosis (SCID) and found to be reliable and valid. For depressive disorders, the MINI showed a sensitivity and specificity of 92%, Kappa 0.77, positive predictive value (PPV) 74%, negative predictive value (NPV) 98% and accuracy of 92% [26].

Adults selected to be included in the validation study sample were invited to receive a home visit from a mental health professional (psychiatry, psychologist or psychiatric resident), all previously trained in the application and interpretation of the gold standard instrument. Training was carried out through seminars and role plays of the MINI application, with the consultant clarifying questions in each session (total duration of 30 h).

The presence of MDE was considered as a gold standard diagnostic. All individuals considered positive for MDE answered an additional group of questions regarding other possible causes for the symptoms, such as direct effects of substances, the presence of organic disorder or other medical illness, the presence of psychotic symptoms or if the symptoms would be better explained by reaction to bereavement, and in this case MDE diagnosis was discarded. Mental health professionals were blinded to participants’ EPDS scores.

Interviews with mental health professionals (gold standard) were performed on average 24 days after the application of the EPDS (minimum interval of 0 days, maximum of 93 days and median of 17 days).

### Data analysis

For each EPDS cutoff point, we calculated the sensitivity or true positive rate (proportion of individuals with MDE according to MINI criteria that were correctly identified by EPDS), specificity or true negative rate (proportion of individuals without MDE according to the gold standard correctly identified as such by EPDS), PPV (proportion of true positives among all positives identified by the EPDS) and NPV (proportion of true negatives among all those who scored negative by EPDS). We calculated 95% confidence intervals for each of these parameters.

We used Youden’s index as a criterion for choosing the “optimal” threshold value for the EPDS test, the threshold value for which the value of [sensitivity + specificity −1] is maximized.

Criterion validity was assessed by receiver operating characteristic (ROC) curves. The ROC curve is a plot of the sensitivity versus [1-specificity] over all possible threshold values of the test being validated. The EPDS point showing simultaneously the highest sensitivity and specificity was also evaluated using the ROC curve. EPDS’s accuracy (proportion of results, both positive and negative, correctly identified by the EPDS) was estimated by the area under the ROC curve.

In order to identify how the EPDS would perform among populations with different depression prevalence rates we performed simulations with different prevalence rates and calculated the PPV based on the sensitivity and specificity obtained for the EPDS at the cutoff points most commonly used internationally [27].

Analyses were conducted for the entire sample and separately for men and women. All analyses were performed using Stata® version 12.0 sofware.

Ethical approval for both the main research and the validation study was granted by the Research Ethics Committee of the Federal University of Pelotas School of Medicine (protocols 77/2011 and 14/2012, respectively). All respondents signed a consent form prior to data collection. Individuals who were at risk of suicide or had severe symptoms of depression were home visited by mental health providers and/or were referred to mental health care services.

## Results

Of 533 individuals selected for the gold standard interview, we had a participation rate of 84% (n = 447). There were 29 refusals, 51 individuals couldn’t be found at home after at least three attempts and six moved out of the city.

Socio-demographic characteristics of the sample are presented in Table 1. A total of 191 men and 256 (57.3%) women were included in the study. Regarding socio-demographic variables, 54.8% had 9 or more years of schooling, 42.3% were 40–59 years old, 76.5% self-reported their skin colour as White, 34.5% were single or not living with a partner and 58.8% were currently employed. Concerning behavioural variables 23.5% were smokers and 41.8% reported alcohol consumption in the month preceding the interview. Individuals lost to the gold standard interview were similar to those who were interviewed in all investigated characteristics (Table 1).

**Table 1:** **Characteristics of the sample included in the validation of the Edinburgh Postnatal Depression Scale (n = 477) and losses (n = 86), Pelotas, 2012**

**Variables**	**Sample**	**Losses**	**p-value** ^**a**^
	**n (%)**	**n (%)**	
Female	256 (57.3)	47 (54.6)	0.65
Schooling (years)			0.27
0-4	68 (15.3)	9 (10.6)	
5-8	133 (29.9)	32 (37.6)	
≥ 9	244 (54.8)	44 (51.8)	
Age (years)			0.14
20-39	183 (40.9)	44 (51.2)	
40-59	189 (42.3)	33 (38.3)	
≥ 60	75 (16.8)	9 (10.5)	
White skin colour	342 (76.5)	70 (81.4)	0.32
Single/without partner	154 (34.5)	34 (39.5)	0.36
Working status			0.52
Working	263 (58.8)	52 (60.5)	
Not currently working	164 (36.7)	28 (32.6)	
Never worked	20 (4.5)	6 (7.0)	
Smoking	105 (23.5)	23 (26.7)	0.51
Alcohol use	187 (41.8)	37 (43.0)	0.83

^a^ *x* ^*2*^ test.

EPDS and MINI scale reliability calculated using Cronbach's alpha was 0.8366 and 0.7875, respectively. Cronbach's alpha range calculated by omitting each question of the instrument ranged between 0.8011-0.8460 for EPDS and 0.7429-0.7910 for MINI.

The gold standard interview identified 40 individuals with MDE (32 women and eight men), corresponding to a global prevalence of MDE of 8.9% (CI 95% 6.3 – 11.6).

**Figure 1:**
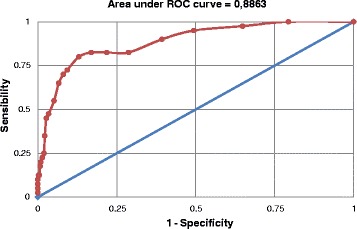
**Receiver operator characteristic curve for the performance of the Edinburgh Postnatal Depression Scale compared to an interview with a mental health Professional using Mini International Neuropsychiatric Interview (gold standard) for the diagnosis of major depressive episode.** Pelotas, 2012 (n=447).

Table 2 shows sensitivity, specificity, PPV, NPV and accuracy for each of the EPDS cutoff points compared to the gold standard interview. As expected, sensitivity decreased progressively as the cutoff point increased, with a more marked decrease between the ≥4 and ≥5 cutoff points (from 90.0% to 82.5%). In contrast, specificity between these two cutoff points increased from 60.7% to 71.3%. Both Youden’s index and the cutoff point of maximum sensitivity and specificity according to the ROC curve (Figure 1) indicate the ≥8 cutoff point as the most suitable for identifying individuals at increased risk of having MDE among this population. A total of 85 individuals (19.0%; 15.5 – 23.0%) scored ≥8 in the EPDS. Sensitivity at this point was 80.0% (64.4 – 90.9%) and specificity of 87.0% (83.3 – 90.1%). The PPV and NPV were 37.6% (27.4 – 48.8%) and 97.8% (95.7 – 99.0%), respectively. The positive likelihood ratio at this point was 6.1 (4.6 – 8.3) and the area under the ROC curve indicates an accuracy of the EPDS of 88.6%.

**Table 2:** **Number of positive individuals (%) according to the Edinburgh Postnatal Depression Scale (EPDS), sensitivity, specificity, PPV, NPV, accuracy ( 95% confidence intervals) for different EPDS cutoff points compared to the gold standard (International Neuropsychiatric Interview) and Youden**’**s index, adult population from Pelotas (n = 447), 2012**

**Cutoff points**	**N (%)**	**Sensitivity (CI 95%) %**	**Specificity (CI 95%) %**	**PPV %**	**NPV %**	**Accuracy %**	**Youden’** s index (*)
≥ 1	363 (81.2)	100.0 (91.2 – 100.0)	20.6 (16.8 – 24.9)	11.0 (8.0 – 14.7)	100.0 (95.7 – 100.0)	60.3 (58.4 – 62.3)	0.206
≥ 2	303 (67.8)	97.5 (86.8 – 99.9)	35.1 (30.5 – 40.0)	12.9 (9.3 – 17.2)	99.3 (96.2 – 100.0)	66.3 (62.9 – 70.0)	0.326
≥ 3	239 (53.5)	95.0 (83.1 – 99.4)	50.6 (45.6 – 55.6)	15.9 (11.5 – 21.2)	99.0 (96.6 – 99.9)	72.8 (68.6 – 77.0)	0.456
≥ 4	196 (43.8)	90.0 (76.3 – 97.2)	60.7 (55.8 – 65.5)	18.4 (13.2 – 24.5)	98.4 (96.0 – 99.6)	75.3 (70.1 – 80.6)	0.507
≥ 5	150 (33.6)	82.5 (67.2 – 92.7)	71.3 (66.6 – 75.6)	22.0 (15.7 – 29.5)	97.6 (95.2 – 99.0)	76.9 (70.5 – 83.2)	0.538
≥ 6	122 (27.3)	82.5 (67.2 – 92.7)	78.1 (73.8 – 82.1)	27.0 (19.4 – 35.8)	97.8 (95.6 – 99.1)	80.3 (74.0 – 86.6)	0.606
≥ 7	102 (22.8)	82.5 (67.2 – 92.7)	83.0 (79.0 – 86.6)	32.4 (23.4 – 42.3)	98.0 (95.9 – 99.2)	82.8 (76.5 – 89.0)	0.655
≥ 8	85 (19.0)	80.0 (64.4 – 90.9)	87.0 (83.3 – 90.1)	37.6 (27.4 – 48.8)	97.8 (95.7 – 99.0)	83.5 (77.0 – 90.0)	0.670
≥ 9	67 (15.0)	72.5 (56.1 – 85.4)	90.7 (87.4 – 93.3)	43.3 (31.2 – 56.0)	97.1 (94.9 – 98.5)	81.6 (74.4 – 88.7)	0.632
≥10	61 (13.6)	70.0 (53.5 – 83.4)	91.9 (88.8 – 94.4)	45.9 (33.1 – 59.2)	96.9 (94.6 – 98.4)	80.9 (73.6 – 88.3)	0.619
≥ 11	53 (11.9)	65.0 (48.3 – 79.4)	93.4 (90.5 – 95.6)	49.1 (35.1 – 63.2)	96.4 (94.1 – 98.0)	79.2 (71.6 – 86.8)	0.584
≥ 12	43 (9.6)	55.0 (38.5 – 70.7)	94.8 (92.2 – 96.8)	51.2 (35.5 – 66.7)	99.5 (93.0 – 97.3)	74.9 (67.0 – 82.8)	0.498
≥ 13	33 (7.4)	47.5 (31.5 – 63.9)	96.6 (94.3 – 98.1)	57.6 (39.2 – 74.5)	94.9 (92.4 – 96.8)	72.0 (64.1 – 79.9)	0.441
≥ 14	29 (6.5)	45.0 (29.3 – 61.5)	97.3 (95.2 – 98.6)	62.1 (42.3 – 79.3)	94.7 (92.1 – 96.7)	71.1 (63.3 – 79.0)	0.423
≥ 15	23 (5.1)	35.0 (20.6 – 51.7)	97.8 (95.8 – 99.0)	60.9 (38.5 – 80.3)	93.9 (91.1 – 96.0)	66.4 (58.9 – 73.9)	0.328
≥ 16	18 (4.0)	25.0 (12.7 – 41.2)	98.0 (96.2 – 99.1)	55.6 (30.8 – 78.5)	93.0 (90.2 – 95.2)	61.5 (54.7 – 68.3)	0.230
≥ 17	15 (3.4)	22.5 (10.8 – 38.5)	98.5 (96.8 – 99.5)	60.0 (32.3 – 83.7)	92.8 (90.0 – 95.1)	60.5 (53.9 – 67.1)	0.210
≥ 18	12 (2.7)	20.0 (9.1 – 35.6)	99.0 (97.5 – 99.7)	66.7 (34.9 – 90.1)	92.6 (89.8 – 94.9)	59.5 (53.2 – 65.8)	0.190
≥ 19	10 (2.2)	17.5 (7.3 – 32.8)	99.3 (97.9 – 99.8)	70.0 (34.8 – 93.3)	92.4 (89.6 – 94.7)	58.4 (52.4 – 64.4)	0.168
≥ 20	10 (2.2)	17.5 (7.3 – 32.8)	99.3 (97.9 – 99.8)	70.0 (34.8 – 93.3)	92.4 (89.6 – 94.7)	58.4 (52.4 – 64.4)	0.168
≥ 21	7 (1.6)	12.5 (4.2 – 26.8)	99.5 (98.2 – 99.9)	71.4 (29.0 – 96.3)	92.0 (89.1 – 94.4)	56.0 (50.8 – 61.2)	0.120
≥ 22	6 (1.3)	12.5 (4.2 – 26.8)	99.8 (98.6 – 100)	83.3 (35.9 – 99.6)	92.1 (89.1 – 94.4)	56.1 (50.9 – 61.3)	0.123
≥ 23	4 (0.9)	10.0 (2.8 – 23.7)	100.0 (99.1 – 100.0)	100.0 (39.8 – 100.0)	91.9 (88.9 – 94.2)	55.0 (50.3 – 59.7)	0.100

Note: PPV = positive predictive value; NPV = negative predictive value.

(*) Youden’s índex = [sensitivity + specificity – 1].

**Figure 2:**
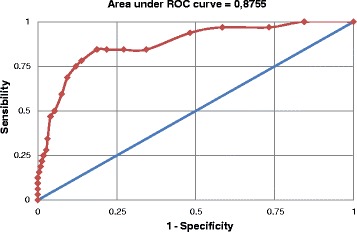
**Receiver operator characteristic curve for the performance of the Edinburgh Postnatal Depression Scale compared to an interview with a mental health Professional using Mini International Neuropsychiatric Interview (gold standard) for the diagnosis of major depressive episode among women.** Pelotas, 2012 (n=256).

EPDS performance among women was very similar to the results of the entire population (Figure 2). The best cutoff point was ≥8 too with values of sensitivity and specificity of 84.4% (67.2 – 94.7%) and 81.3% (75.5 – 86.1%), respectively. The PPV and NPV were 39.1% (27.6 – 51.6%) and 97.3 (93.9 – 99.1%), respectively. The positive likelihood ratio at this cutoff point was 4.5 (3.3 – 6.1) and the area under the curve indicates an accuracy of the EPDS of 87.6%.

**Figure 3:**
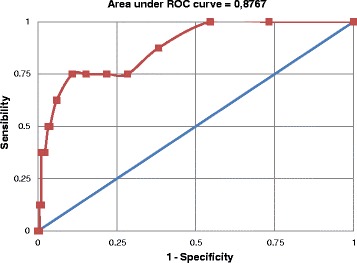
**Receiver operator characteristic curve for the performance of the Edinburgh Postnatal Depression Scale compared to an interview with a mental health Professional using Mini International Neuropsychiatric Interview (gold standard) for the diagnosis of major depressive episode among men.** Pelotas, 2012 (n=191).

Among men, the best cutoff point according Youlden’s index and the ROC curve (Figure 3) was ≥7. Sensitivity at this point was 75.0% (34.9 – 96.8%) and specificity of 89.1% (83.6 – 93.2%). The PPV and NPV were 23.1% (9.0 – 43.6%) and 98.8% (95.7 – 99.9%), respectively. The positive likelihood ratio at this cutoff point was 6.9 (3.9 – 12.2) and the area under the ROC curve indicates an accuracy of the EPDS of 87.7%.

The effect of changes in the prevalence of MDE in the study population was observed in the PPV of EPDS. Table 3 shows the PPV for EPDS cutoff points between 7 and 13 in simulations for populations with different depression prevalence rates. Thus, for instance, if EPDS was administered as a screening test with a cutoff point of ≥8 in a population with a depression prevalence rate of about 10%, the PPV would be 40.6%. In this case, 60% of individuals identified by EPDS as suffering from depression would actually be false-positives. If the prevalence rate of depression in the population increases, the PPV increases as well, however, there still persist a large number of false positives in the population. False positives would be less than 40% using a cutoff of ≥13 in a population with a prevalence rate of depression of 10% and false positive would be less than 25% with a prevalence rate of depression of 20%.

**Table 3:** **Positive predictive values (confidence intervals of 95%) for different Edinburgh Postnatal Depression Scale (EPDS) cutoff points, according to the prevalence of depression in the study population, Pelotas, 2012**

**Cutoff point**	**Positive predictive value**
*EPDS ≥7*	
Prevalence of depression	
5%	20.4 (16.5 – 24.9)
10%	35.1 (29.5 – 41.2)
15%	46.2 (39.9 – 52.6)
20%	54.9 (48.4 – 61.2)
25%	61.9 (55.6 – 67.7)
*EPDS ≥8*	
Prevalence of depression	
5%	24.4 (19.4 – 30.3)
10%	40.6 (33.7 – 47.8)
15%	52.0 (44.7 – 59.3)
20%	60.6 (53.3 – 67.4)
25%	67.2 (60.4 – 73.3)
*EPDS ≥9*	
Prevalence of depression	
5%	29.0 (22.2 – 36.9)
10%	46.3 (37.6 – 55.2)
15%	57.8 (48.9 – 66.2)
20%	66.0 (57.6 – 73.5)
25%	72.1 (64.4 – 78.7)
*EPDS ≥10*	
Prevalence of depression	
5%	31.2 (23.6 – 40.0)
10%	49.0 (39.5 – 58.5)
15%	60.4 (50.9 – 69.1)
20%	68.3 (59.5 – 76.0)
25%	74.2 (66.2 – 80.9)
*EPDS ≥11*	
Prevalence of depression	
5%	34.0 (25.1 – 44.2)
10%	52.1 (41.5 – 62.6)
15%	63.4 (52.9 – 72.7)
20%	71.0 (61.5 – 79.0)
25%	76.6 (68.0 – 83.4)
*EPDS ≥12*	
Prevalence of depression	
5%	35.9 (25.3 – 48.1)
10%	54.2 (41.8 – 66.2)
15%	65.3 (53.2 – 75.7)
20%	72.7 (61.7 – 81.5)
25%	78.0 (68.3 – 85.4)
*EPDS ≥13*	
Prevalence of depression	
5%	42.1 (28.3 – 57.2)
10%	60.5 (45.5 – 73.8)
15%	70.9 (57.0 – 81.8)
20%	77.5 (65.2 – 86.4)
25%	82.2 (71.5 – 89.4)

## Discussion

The present study showed that the EPDS is a suitable test for screening MDE among adults from the community and living in an urban middle-sized city like Pelotas. A screening test should focus on the sensitivity, in order to maximize the number of individuals who could be in need of health care. Using the cutoff point of ≥8 and with a prevalence of MDE in the population of about 10%, around 20% of individuals will be EPDS positive and when referred to mental health services, half will be diagnosed with MDE. To be used as a diagnostic test, the EPDS cutoff point needs to be higher, with a suggested cutoff point of ≥13, favouring the specificity instead of the sensitivity.

The EPDS was originally devised for the identification of postpartum depression disorders for use in clinical and research settings [16]. The clinical and epidemiological value of the scale has been confirmed by several validation studies carried out among women in the antenatal and postpartum period all around the world [19,22,23,25,28-30]. In Brazil, the EPDS was previously validated among women showing good psychometric properties [17,18,24,31]. However, in the only population-based validation study carried out in the postpartum period made in Brazil, sensitivity, specificity and PPVs for all cutoff points were below those previously reported, especially when compared to studies from high-income countries [24]. The EPDS was also validated among men in the postpartum period [21,32,33], having been found more accurate than the Beck Depression Inventory (BDI) and the Patient Health Questionnaire – Depression Module (PHQ-9) among Chinese men [33].

Outside the antenatal and postpartum period, when the scale can be referred to as Edinburgh Depression Scale (EDS) [34], few validation studies were identified [34-36]. Cox et al. [34] validated the EPDS among 136 women recruited from general practice registers that were non-pregnant and who had had no births in the previous year. The authors reported an acceptable EPDS performance for screening major depression with a cutoff point of 12/13 (sensitivity, specificity and PPV of 88%, 80% and 21%, respectively) using Goldberg’s Clinical Interview Schedule as the gold standard. The EPDS was also validated in a large community sample of 951 menopausal women by Becht et al. [35] using the Research Diagnostic Criteria as the gold standard. The EPDS was chosen to be applied in this population of women because it doesn’t include somatic symptoms related to menopause, such as sleeping problems and sexual dysfunction. With a cutoff point of 12 points or higher, the EPDS showed appropriate psychometric characteristics for screening major depressive disorder (sensitivity, specificity and PPV of 88%, 85% and 40%, respectively). The advantage of the EPDS of not containing any somatic-type symptom was also considered by Lloyd-Williams et al. [36] in their study of 100 patients (55 females and 44 males) with advanced metastatic cancer receiving palliative care and using the Present State Examination (semi structured psychiatric interview) as gold standard. With a cutoff of 13 or higher, the EPDS showed similar sensitivity and PPV to the study performed among women at menopausal age (sensitivity, specificity and PPV of 81%, 79% and 53%, respectively).

In our study with a cutoff score of ≥8, the percentage of correctly identified people with MDE was similar to those of the three other validation studies performed outside the postpartum period (all sensitivities fell within the 95% confidence interval of our estimative) [34-36]. The PPV values of our study for different cutoff points were not very high, but still almost two-times higher than the values observed in Cox et al. study [34], probably due to the small sample size of their study.

The best cutoff point for screening depression in our study (≥8) was lower than the cutoff point recommended for screening MDE among those studies performed outside the postpartum period [34-36]. In addition, for a 12/13 cutoff, EPDS sensitivity dropped to less than 50% and its specificity almost reach 95%, values that could be found in a diagnostic test and not suitable for screening. The EPDS cutoff scores vary from culture-to-culture and recommendations have been made to validate the instrument before applying it for screening to a population [37].

In our study, the EPDS score values were more concentrated among men and very few of them (only eight) were diagnosed as depressed by our gold standard. Better EPDS performance among women than among men was seen in a previous study performed in the postpartum period [38]. Our literature review identified very few validation studies of the EPDS among people from the general population outside the prenatal or postpartum period. In addition, difficulties have been reported to reach the necessary number of men in the study to calculate EPDS’s psychometric properties in this group [39].

Our study was the first EPDS validation study for screening MDE among people from the general population and outside the postpartum period in Brazil. The EPDS has a long track record of being routinely administered not only to mothers but also fathers in the postnatal period. Given the ease of administration and acceptability as well as its good psychometric properties the EPDS could be a useful tool to screen depression in the adult population in both research and clinical settings. Administration of the scale as an interview was appropriate for the cultural characteristics of people included in the sample. However, it is possible that this strategy could have biased the respondents’ answers due to social desirability effect of face-to-face interview and our results could not be generalized to situations where the EPDS is self-reported. Among other limitations of the study, we had 16% of individuals that could not undergo the gold standard interview and, though, were not included in the validation sample. They were similar to those included in the sample regarding all socioeconomic, demographic and behavioural characteristics investigated. In addition, the prevalence of EPDS ≥8 among people that failed to be included in the validation sample was similar to those included in the sample (24% versus 19%, respectively, p = 0.250). It looks like the loss of these individuals may not have impaired the sensitivity estimation in the present study. Another limitation was the gap of about 17 days (median) between the EPDS and the gold standard administration. It is possible that depressive symptoms may have changed over this period, mainly because the test was designed to enquire about feelings over the last seven days. However, it is frequently observed that depressive symptoms usually persist for weeks or even months [40]. Finally, care should be taken when deciding to compare the results of previously EPDS validation studies with our study. Methodological issues such as the prevalence of depression in the target population, how cases were defined by the instrument chosen as gold standard, the design of the study and the specific characteristic of the population where the validation was performed, are all characteristics that could affect the EPDS’s psychometric properties.

## Conclusions

In conclusion, the EPDS was shown to be suitable for screening MDE among adult populations living in the urban area of medium-sized cities and with similar cultural characteristics to the population where the present study was conducted. The EPDS can easily be applied by trained interviewers, not necessarily psychiatrics or psychologists which are in short supply in low and middle-income countries, and this continues to be the main advantage of the instrument. Even though the final diagnostic of depression can only be confirmed through an interview with a mental health professional, the EPDS could be used to monitor MDE prevalence in the community. More studies are needed in men, where EPDS psychometric properties of the test could not be tested properly.

## Abbreviations

EPDS: Edinburgh postnatal depression scale
MDE: Major depressive episode
MINI: Mini international neuropsychiatric interview
DSM-IV: Diagnostic and statistical manual of mental disorders – IV revision
ICD-10: International statistical classification of diseases and related health problems – 10th revision
ROC: Receiver operator characteristic
PHQ-9: Patient health questionnaire
EDS: Edinburgh depression scale
PPV: Positive predictive value
NPV: Negative predictive value
